# Transcriptome analysis of green and purple fruited pepper provides insight into novel regulatory genes in anthocyanin biosynthesis

**DOI:** 10.7717/peerj.16792

**Published:** 2024-01-17

**Authors:** Huaqiang Tan, Liping Li, Manman Tie, Ronghai Lu, Shaokun Pan, Youwan Tang

**Affiliations:** 1 Chengdu Academy of Agriculture and Forestry Sciences, Chengdu, Sichuan, China; 2 Agricultural and Rural Bureau of Lushan County, Yaan, Sichuan, China

**Keywords:** Pepper, Transcriptome, Purple, Anthocyanin, Transcription factor

## Abstract

**Background:**

Pepper (*Capsicum annuum* L.) is a valuable horticultural crop with economic significance, and its purple fruit color is attributed to anthocyanin, a phytonutrient known for its health-promoting benefits. However, the mechanisms regulating anthocyanin biosynthesis in pepper have yet to be fully elucidated.

**Methods:**

RNA sequencing (RNA-seq) was utilized to analyze the transcriptome of fruits from three purple-fruited varieties (HN191, HN192, and HN005) and one green-fruited variety (EJT) at various developmental stages. To determine the relationships between samples, Pearson correlation coefficients (PCC) and principal component analysis (PCA) were calculated. Differential expression analysis was performed using the DESeq2 package to identify genes that were expressed differently between two samples. Transcription factors (TF) were predicted using the iTAK program. Heatmaps of selected genes were generated using Tbtools software.

**Results:**

The unripe fruits of HN191, HN192, and HN005, at the stages of 10, 20, and 30 days after anthesis (DAA), display a purple color, whereas the unripe fruits of variety EJT remain green. To understand the molecular basis of this color difference, five transcriptome comparisons between green and purple fruits were conducted: HN191-10 *vs* EJT-10, HN191-20 *vs* EJT-20, HN191-30 *vs* EJT-30, HN192-30 *vs* EJT-30, and HN005-30 *vs* EJT-30. Through this analysis, 503 common differentially expressed genes (DEGs) were identified. Among these DEGs, eight structural genes related to the anthocyanin biosynthesis pathway and 24 transcription factors (TFs) were detected. Notably, one structural gene (MSTRG.12525) and three TFs (T459_25295, T459_06113, T459_26036) exhibited expression patterns that suggest they may be novel candidate genes involved in anthocyanin biosynthesis. These results provide new insights into the regulation of anthocyanin biosynthesis in purple pepper fruit and suggest potential candidate genes for future genetic improvement of pepper germplasm with enhanced anthocyanin accumulation.

## Introduction

Anthocyanins are natural pigments responsible for a wide range of colors—from orange and red to purple and blue—in flowers, seeds, fruits, and vegetative tissues (Tanaka, Sasaki & Ohmiya, 2008). These flavonoids have important biological functions in plants, such as attracting insects to spread seeds, protecting against photo-oxidative damage, and improving resistance to stresses (Gould, 2004; Steyn et al., 2002; Chalker-Scott, 1999). More importantly, anthocyanins have antioxidant properties that benefit human health and may help prevent chronic and degenerative illnesses like cancer and cardiovascular disease (Reddy, Alexander-Lindo & Nair, 2005; Lamy et al., 2007; De Pascual-Teresa, Moreno & García-Viguera, 2010). As a result, the biosynthesis of anthocyanin becomes a focus of interest for the genetic improvement of crops.

The biosynthesis of anthocyanin has been extensively studied in various plants and is a branch pathway of flavonoid metabolism, involving two groups of genes: structural and regulatory (Gonzali, Mazzucato & Perata, 2009). Structural genes encode enzymes that directly participate in anthocyanin biosynthesis, such as chalcone synthase (CHS), chalcone isomerase (CHI), flavanone 3-hydroxylase (F3H), flavonoid 3′5′-hydroxylase (F3′5′H), dihydroflavonol 4-reductase (DFR), anthocyanidin synthase (ANS), and flavonoid 3-O-glucosyltransferase (UFGT) (Holton & Cornish, 1995; Zhang et al., 2015). *CHS*, *CHI* and *F3H* are categorized as early biosynthetic genes (EBGs), while *F3′H*, *F3′5′H*, *DFR*, *ANS*, and *UFGT* are classified as late biosynthetic genes (LBGs) (Chen et al., 2022). Meanwhile, regulatory genes encode transcription factors (TFs) that control the expression of structural genes. Three main types of TFs have been identified, including my elob lastosis (MYB), basic helix-loop-helix (bHLH), and WD40-repeat proteins (Zhang, Butelli & Martin, 2014), which can form a regulatory complex called MYB-bHLH-WD40 (MBW) and bind to the promoters of structural genes, especially LBGs, leading to their transcriptional activation and expression (Stommel et al., 2009; Petroni & Tonelli, 2011).

Pepper (*Capsicum annuum* L.) is among the most commonly cultivated vegetable crops worldwide. The unripe pepper fruit can exhibit various colors such as dark green, green, light green, purple, yellow, or ivory (Liu et al., 2020b). The purple color arises from the accumulation of anthocyanins (Lightbourn et al., 2008). It was initially reported that a gene called gene *A* is responsible for the purple fruit color in pepper (Peterson, 1959). This gene *A* was subsequently isolated from pepper and found to encode an R2R3 MYB transcription factor similar to Anthocyanin2 in Petunia (Borovsky et al., 2004). Overexpression of this *CaAn2* gene in *arabidopsis* and tobacco plants resulted in a purple phenotype with increased anthocyanin content (Jung et al., 2019). Further investigation revealed that a non-long terminal repeat (non-LTR) retrotransposon was inserted in the promoter region of *CaAn2* in purple *C. annuum* plants. This retrotransposon may activate the expression of *CaAn2* by recruiting transcription factors at the 3′ UTR (Jung et al., 2019). Additionally, genetic mapping and transcriptome research have aimed to identify more genes involved in anthocyanin biosynthesis. Liu et al. (2020a) employed a map-based cloning strategy to fine-map a structural gene (*Capana10g001978*) responsible for anthocyanin biosynthesis, while Wang et al. (2018) utilized a combination of specific length amplified fragment sequencing (SLAF-seq) and bulked segregant analysis (BSA) to identify 12 candidate genes associated with anthocyanin accumulation. Furthermore, transcriptome analysis identified several genes, including *CaANT1*, *CaANT2*, *CaAN1*, and *CaTTG1*, involved in anthocyanin accumulation in the purple pepper fruit variety No. Co62 (Tang et al., 2020). A combined analysis of metabolome and transcriptome identified ten highly expressed genes encoding transcription factors in the purple fruit variety C1-P, including 2 WD (WD68 and WD44), 1 bHLH (bHLH143), and 1 MADS-box protein (AGL16) (Liu et al., 2020b). Moreover, Meng et al. (2022) identified 59 unigenes, including 7 enzymes and 8 transcription factors, as candidate genes involved in anthocyanin biosynthesis in the purple fruit varieties L29 and L66.

Due to their attractive color, high antioxidant capacity, and positive effects on shelf-life, there is an increasing interest in uncovering the mechanism of anthocyanin metabolism in Solanaceous vegetables such as pepper, eggplant, tomato and potato. Current knowledge on anthocyanins in the Solanaceous vegetables has been reviewed, including biochemistry and biological function of anthocyanins, as well as their genetic and environmental regulation (Liu et al., 2018b). Despite the abundance of literature on anthocyanins in Solanaceae, the transcriptional regulation of anthocyanin biosynthesis in Capsicum remains to be clearly elucidated (Tang et al., 2020). The aim of this study was to investigate the transcriptome dynamics of three purple-fruited pepper varieties (HN191, HN191, HN005) and a green-fruited variety (EJT) across different stages of development using RNA-seq, thus gaining further insight into the genes and regulatory networks involved in anthocyanin biosynthesis in purple pepper fruit.

## Materials & Methods

### Plant materials

Four pepper varieties, HN191, HN192, HN005 and EJT were grown in the experimental field of Chengdu Academy of Agriculture and Forestry Sciences (Chengdu, Sichuan, China). Erjingtiao (EJT) is a famous local pepper variety in Sichuan Province with a long cultivation history. It is characterized by its thin skin, thick flesh, delightful fragrance, moderate spiciness, and high nutritional value. This pepper is an essential ingredient in authentic Sichuan cuisine. The unripe fruits are green, while the ripe ones turn red. It is a very representative green fruit pepper material. HN191, HN192, and HN005 are inbred lines developed by our research group. These lines exhibit purple unripe fruits that transform into red mature fruits. In 2020, they were officially recognized by the Sichuan Provincial Non-Major Crops Committee.

Fruits from HN191 and EJT were harvested at 10, 20, 30, and 60 DAA, while fruits from HN192 and HN005 were harvested at 30 and 60 days after anthesis (DAA). For each variety and developmental stage, we collected three biological replicates and stored them at −80 °C for further experiments.

### Assessment of anthocyanin

Anthocyanin mixtures from 30 DAA fruits of four pepper varieties (HN191, HN192, HN005, and EJT, respectively) were extracted and measured according to the agricultural standard of China (NY/T 2640-2014). The anthocyanin standard included delphinidin, cyanidin, petunidin, pelargonidin, peonidin, and malvidin, which were dissolved in methanol with 10% hydrochloric acid.

### Microscope observation

Fresh fruits of HN191 at 30 DAA were cut to create free-hand sections and observed under a microscope (BX41; Olympus, Tokyo, Japan).

### RNA extraction and sequencing

The pepper fruit samples that had been stored were removed from the −80 °C ultra-low temperature freezer and promptly placed into a foam box containing liquid nitrogen. Subsequently, the samples were taken out from the foam box and ground into powder using sterilized mortar and pestle in liquid nitrogen. The powdered samples were then transferred into 1.5 ml RNase-free microtubes (Corning Incorporated, Corning, NY, USA) immediately. Afterwards, total RNA was extracted using the RNAprep Pure extraction kit (Tiangen Biotech Co, Ltd., Beijing, China) in accordance with the manufacturer’s instructions. The purity and integrity of the RNA samples were evaluated using the Agilent Bioanalyzer 2100 system (Agilent Co., Ltd., Beijing, China). The sequencing library of all samples were constructed, and sequenced on the DNBSEQ-T7 platform in China National GeneBank (CNGB).

### Data analysis

The adaptor and low-quality reads were first removed using Fastp (Chen et al., 2018). The resulting clean reads were then mapped to the pepper genome (Genbank assembly accession: GCA_000512255.2) using HISAT2 software (Kim, Langmead & Salzberg, 2015; Kim et al., 2014). To calculate the FPKM (fragments per kilobase of transcript per million mapped reads) for each gene, StringTie and Ballgown software were employed (Pertea et al., 2016).

The correlation between different samples was analyzed using the Pearson Correlation Coefficient (PCC) and Principal Component Analysis (PCA), which were implemented in the Factoextra and FactoMineR packages in R software.

For the differential expression analysis between samples, DESeq2 software was utilized (Varet et al., 2016). Genes with —log2Fold Change— ≥ 1 and a False Discovery Rate (FDR) < 0.05 were considered as differentially expressed genes (DEGs). The iTAK program was used to predict transcription factors from the DEGs (Zheng et al., 2016). Additionally, heatmaps of selected genes were generated using Tbtools software (Chen et al., 2020).

### qRT-PCR analysis

The qPCR assay was configured following the recommendations of ‘The MIQE guidelines’ (Bustin et al., 2009). Ten structural genes and eleven regulatory genes (transcription factors, TFs) were selected to validate the RNA-seq results. Primers for qRT-PCR were designed using the Primer3 online software (http://bioinfo.ut.ee/primer3-0.4.0/) and synthesized by Sangon Biotech Co., Ltd., Shanghai, China (Table S1). For the cDNA synthesis, 1 µg of total RNA was reverse transcribed using the PrimeScript™ RT reagent Kit (Takara Bio Inc., San Jose, CA, USA), following the manufacturer’s protocol. Quantitative RT-PCR was performed on a CFX96 Real-Time PCR system (Bio-Rad Laboratories Inc., Hercules, CA, USA) using TB Green^®^ Premix Ex Taq™ II (Takara Bio Inc., Beijing, China). The consumables used include RNase-free tips and 8 Strip PCR tubes from Axygen^®^ Brand Products (Corning Incorporated, Corning, NY, USA). The quantification was performed in triplicate using 25 µL reactions. Each reaction included 12.5 µl of TB Green Premix Ex Taq II, 1 µl of each primer (10 µM), 8.5 µl RNase-free water, and 2 µl of 1:5 diluted cDNA. The PCR amplification conditions consisted of an initial denaturation at 95 °C for 30 s, followed by 40 cycles of denaturation at 95 ° C for 5 s and annealing at 60 °C for 30 s. A melting curve was obtained at the end of each PCR by gradually increasing the temperature to 95 °C (increment rates of 0.5 °C/s) after cooling to 65 °C for 5 s. Each gene was analyzed on the same amplification for all samples, so inter-run calibration was not necessary. The data obtained were analyzed by the Bio-Rad CFX Manager software (version 3.0), which generated the raw quantification cycle (Cq) values for each reaction. The relative expression of the selected genes was normalized to a pepper actin gene (GenBank ID: T459_30033) and calculated using the 2-ΔΔCT method. Further qPCR details are supplied in a MIQE checklist table (Table S2).

## Results

### Phenotype analysis

**Figure 1:**
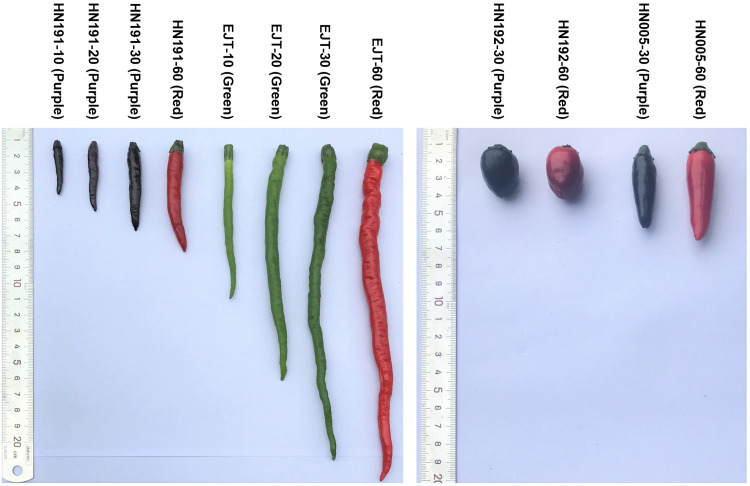
Fruit samples of four pepper varieties at different developmental stages.

**Figure 2:**
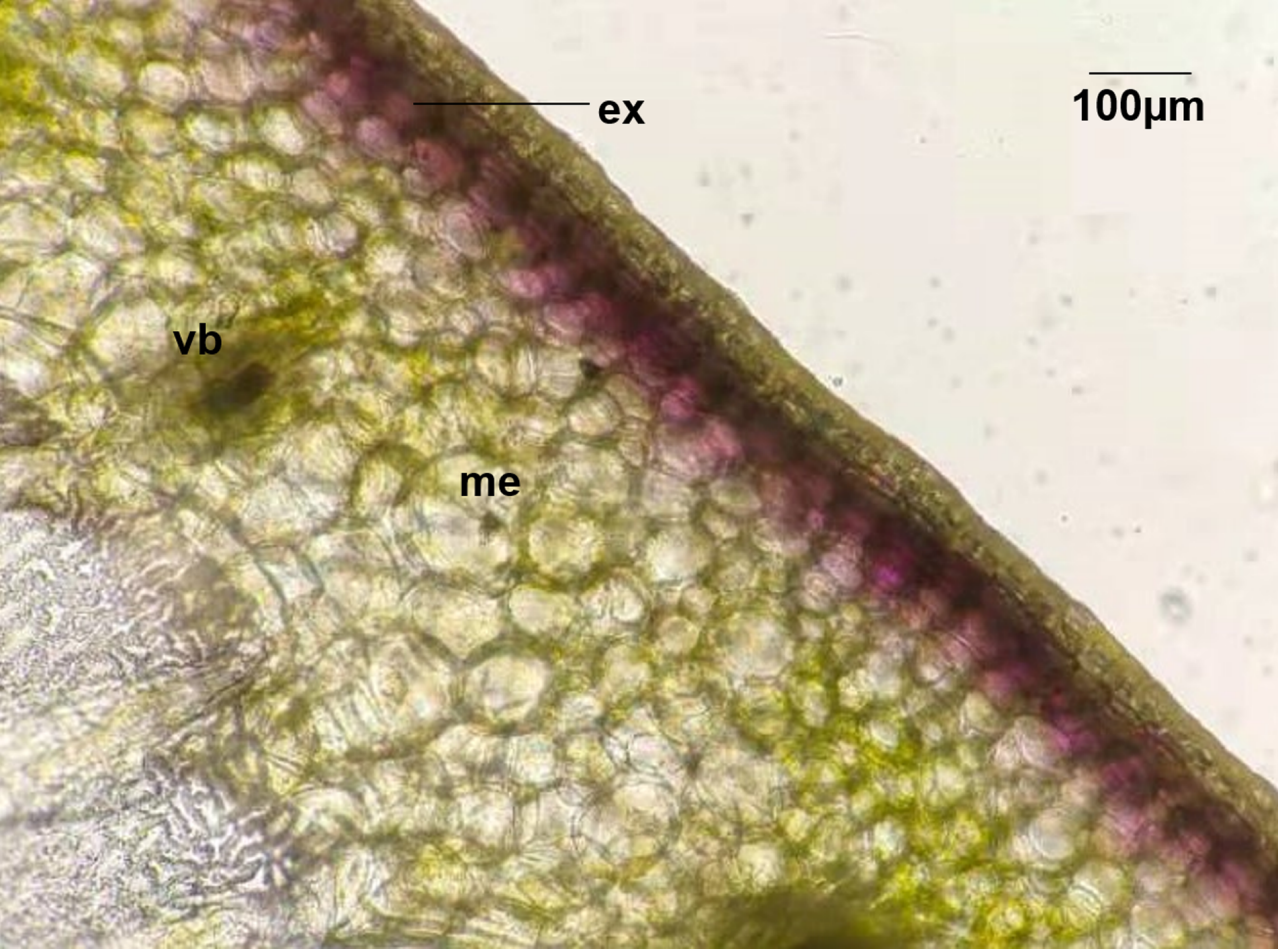
Distribution of Anthocyanins in HN191 fruits at 30 days after anthesis (DAA). Me, mesocarp; Vb, vascular bundle; Ex, exocarp.

In this study, four pepper varieties (HN191, HN192, HN005, and EJT) were selected as research materials. The unripe fruits of HN191, HN192, and HN005 have a purple color at 10, 20, and 30 days after anthesis (DAA), while the unripe fruits of EJT are green (Fig. 1). However, all four varieties have red ripe fruits at 60 DAA. The result of HPLC analysis showed that delphinidin was the only anthocyanin present in the three purple-fruited varieties (HN191, HN192, and HN005), while the levels of cyanidin, petunidin, pelargonidin, peonidin, and malvidin were below the detection threshold. No anthocyanins were detected in the green-fruited variety EJT. Furthermore, when we examined the fruits under a microscope, we observed that anthocyanins were only accumulated in the exocarp of HN191 fruits at 30 DAA (Fig. 2).

### Global transcriptome results

We performed RNA-seq to investigate the dynamics of the transcriptome in fruit samples obtained from HN191 and EJT at four stages (10, 20, 30, and 60 DAA), as well as fruit samples from HN192 and HN005 at two stages (30 and 60 DAA). We analyzed three independent biological replicates of fruits for each variety and stage, resulting in a total of 36 samples. The extracted RNA exhibited a yield ranging from 14 to 52 µg and an RNA Integrity Number (RIN) ranging from 7.2 to 9.1, indicating relatively high RNA quality (Table S3).

A total of 7.25 billion clean reads, representing 725.48 GB of clean bases, were generated from all samples (Table S4) and were mapped to the pepper genome (Kim et al., 2014) at an average rate of 94.03% (range: 92.80%–94.80%). Power analysis on the transcriptome data was conducted using the RNASeqPower package (Hart et al., 2013). Based on a sample size of three (the number of biological replicates used in this study), the statistical power among different groups ranged from 0.85 to 0.91, with an average value of 0.87 (Table S5).

**Figure 3:**
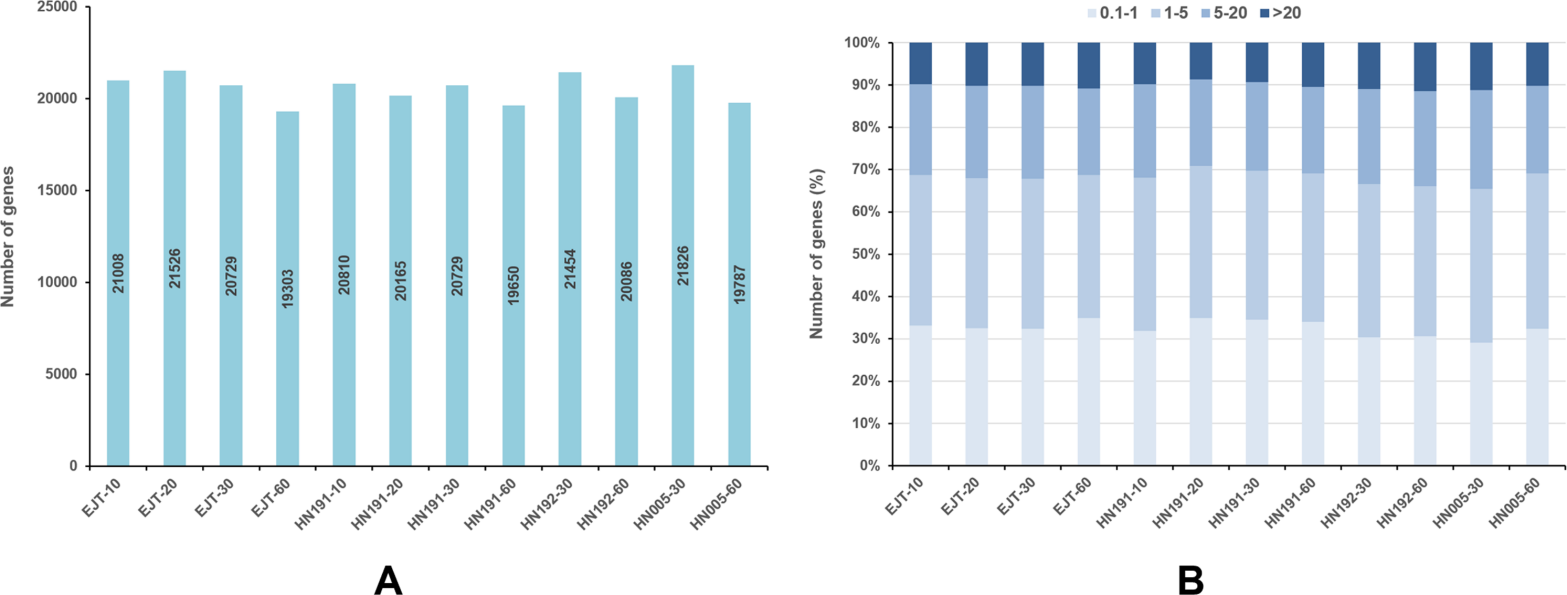
Genes expressed in four pepper varieties at different developmental stages. (A) Genes expressed with FPKM > 1. (B) The fraction of genes with differential expression in EJT and HN191 at 10, 20, 30, and 60 DAA, as well as HN192 and HN005 at 30 and 60 DAA.

For each gene, the FPKM values were calculated, and genes with FPKM >1 were considered expressed. In EJT and HN191 at 10, 20, 30, and 60 DAA, as well as HN192 and HN005 at 30 and 60 DAA, a total of 19,303–21,826 genes were found to be expressed (Fig. 3A). The expression of 9%–11% of genes was very high (FPKM > 20) in the four pepper varieties at different developmental stages (Fig. 3B). Genes with high expression (FPKM 5 to ≤ 20), moderate expression (FPKM 1 to ≤ 5), and low expression (FPKM 0.1 to ≤ 1) accounted for 20%–23%, 34%–37%, and 29%–35% of the total genes, respectively.

### Comparison of global transcriptome between samples

**Figure 4:**
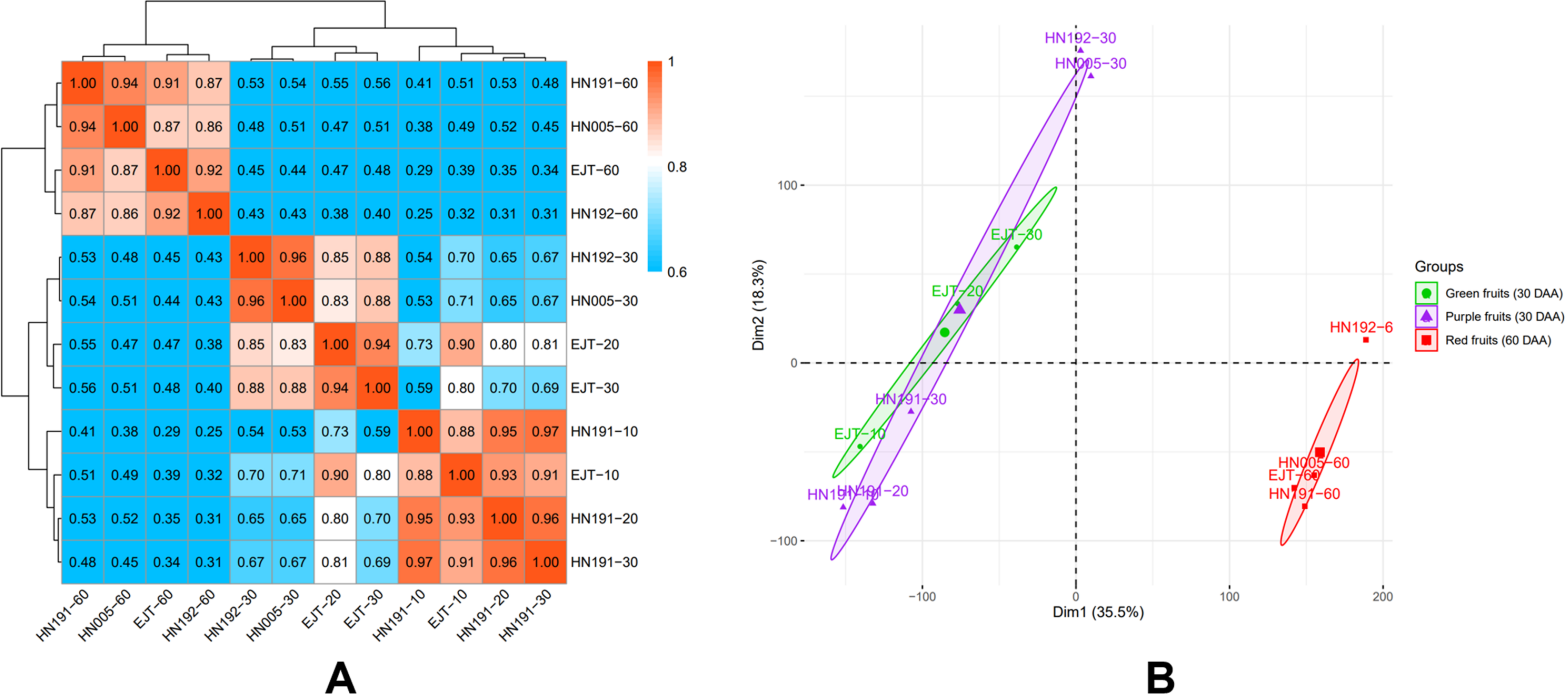
Correlation between transcriptomes of four pepper varieties at different developmental stages. (A) The results of Pearson correlation coefficient analysis. (B) The results of principal component analysis. Ellipses indicate 95% confidence intervals.

The global differences in transcriptomes during fruit development in HN191, HN192, HN005, and EJT varieties were investigated. The mean FPKM values of three biological replicates were used to calculate the Pearson correlation coefficients (PCC) for all samples (Fig. 4A). The PCC values between purple and green unripe fruits ranged from 0.69 to 0.88 (HN191-10 *VS* EJT-10, 0.88; HN191-20 *VS* EJT-20, 0.80; HN191-30 *VS* EJT-30, 0.69; HN192-30 *VS* EJT-30, 0.88; HN005-30 *VS* EJT-30, 0.88). However, for each variety, the PCC values between unripe fruits (10, 20, 30 DAA) and ripe fruits (60 DAA) were relatively low, measuring below 0.6.

Additionally, principal component analysis (PCA) was performed (Fig. 4B). The distances between samples in the plot indicate the similarity of their transcriptional programs. Samples of unripe fruits, both green and purple, were located on the left side of the plot, indicating that the transcriptional activity of purple and green unripe fruits is similar despite the difference in fruit color. On the other hand, samples of ripe fruits clustered on the right side, suggesting that unripe and ripe fruits are more likely to exhibit different transcriptomes and functions/activities, which aligns with the Pearson correlation coefficient results.

### DEG analysis

In order to investigate genes associated with anthocyanin biosynthesis in pepper, our focus was on analyzing the differentially expressed genes (DEGs) between five comparisons of purple and green fruit varieties at 10, 20, and 30 days after anthesis (DAA). These comparisons included HN191 and EJT at 10 DAA, HN191 and EJT at 20 DAA, HN191 and EJT at 30 DAA, HN192 and EJT at 30 DAA, and HN005 and EJT at 30 DAA.

**Figure 5:**
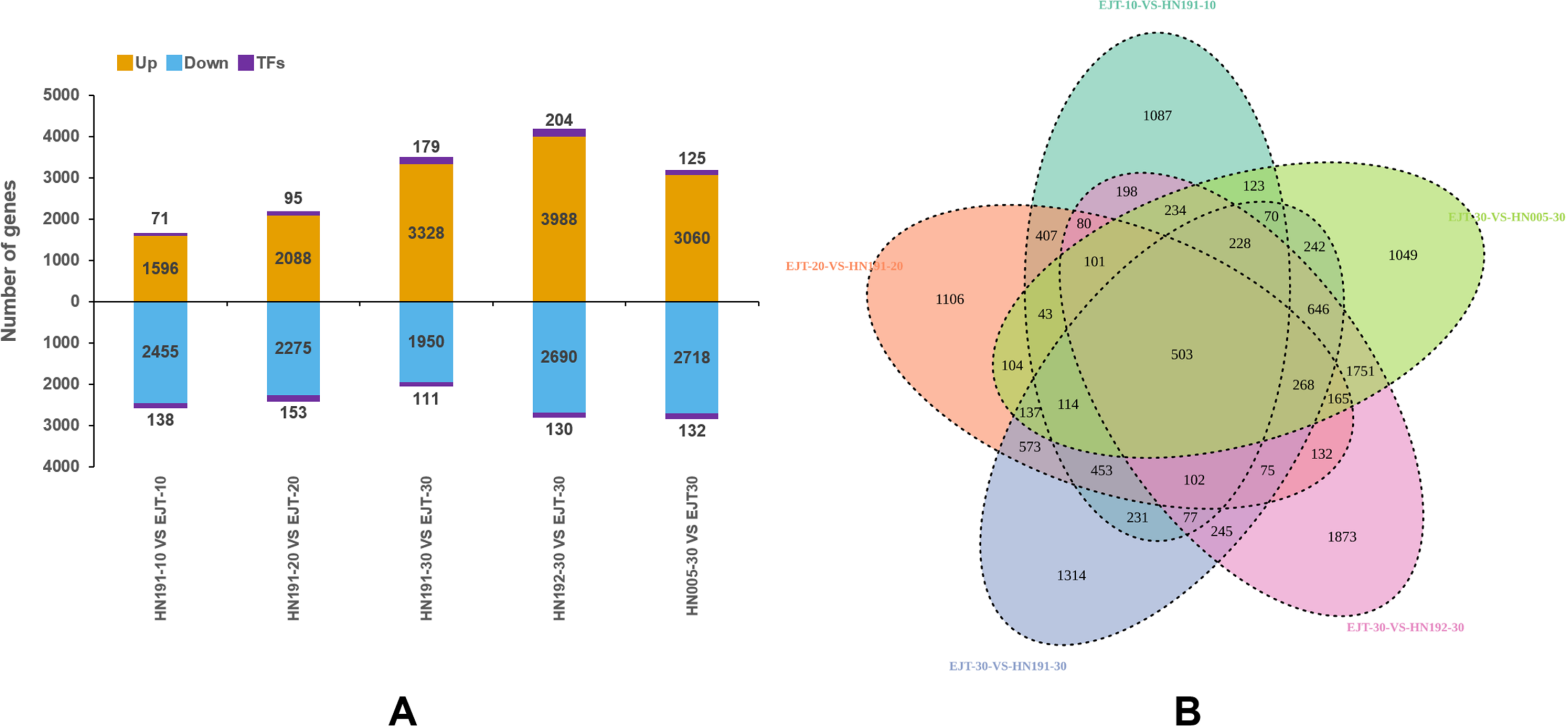
Statistics of differential gene expression in purple fruit varieties (HN191, HN192, HN005) compared with the green fruit variety (EJT) at different stages. (A) The statistics of diûerentially expressed genes. Orange, blue, and purple indicate upregulated genes, downregulated genes, and transcription factors (TFs), respectively. (B) Venn plot of differentially expressed genes between the five comparisons.

In total, we identified 14,734 upregulated genes, which included 674 genes encoding transcription factors (TFs), and 12,752 downregulated genes, which included 664 TF-encoding genes (Fig. 5A). The highest number of differentially expressed genes (7,012) was observed between HN192 and EJT at 30 DAA (HN192-30 *VS* EJT-30), followed by HN005 and EJT at 30 DAA (HN005-30 *VS* EJT-30). Conversely, the smallest number of differentially expressed genes (4,260) was found between HN191 and EJT at 10 DAA (HN191-10 *VS* EJT-10).

### Common DEGs between purple and green fruits

The key distinguishing factor among these five comparisons is the fruit color, with green fruits being compared to purple fruits. Venn diagrams of the DEGs in each of the five comparisons were plotted using Venny2.1 (Fig. 5B). Among these comparisons, a total of 503 DEGs were identified that were common across all five, indicating their potential involvement in the biosynthesis of anthocyanin in pepper fruit.

### Structure genes identified from common DEGs

**Figure 6:**
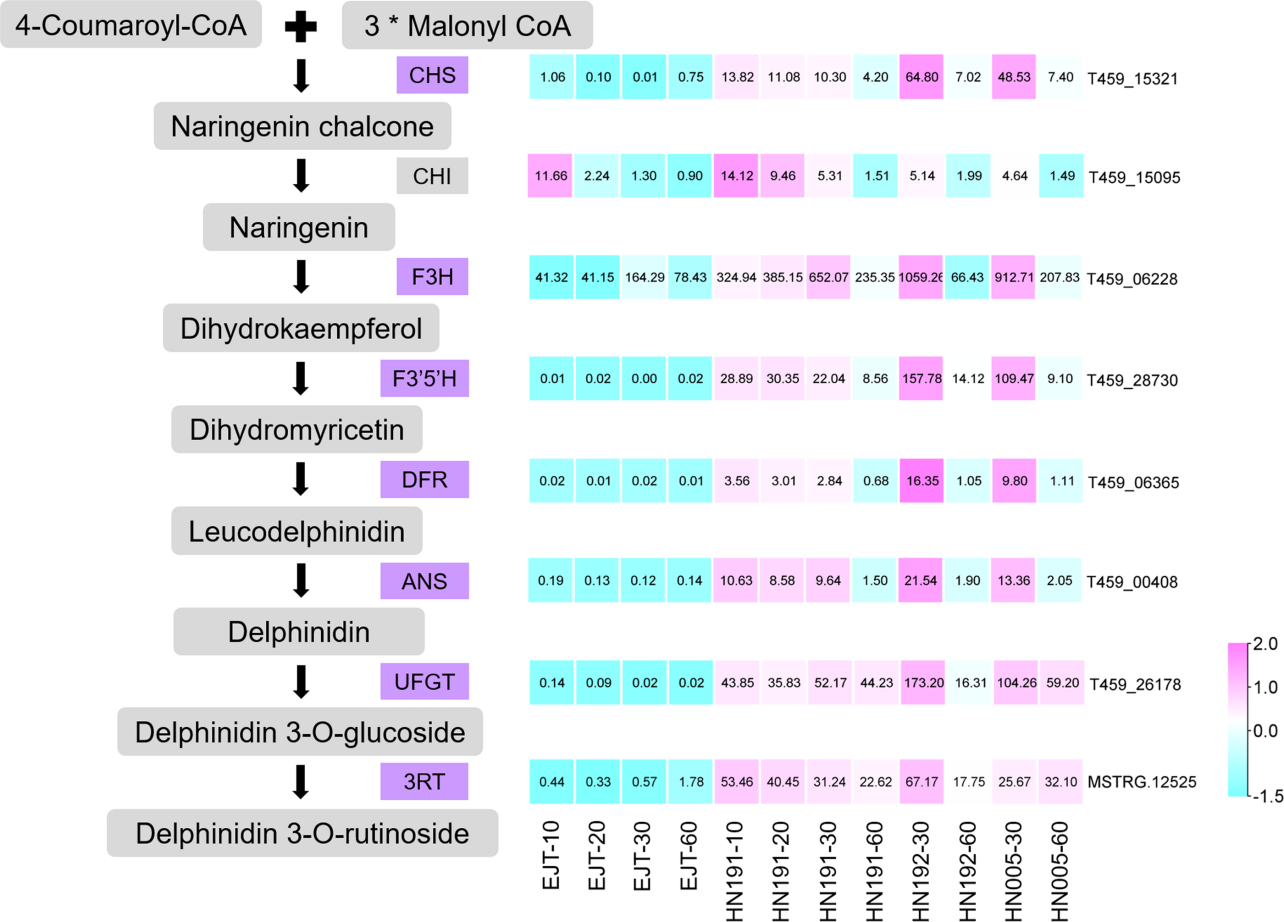
Structural genes of anthocyanin biosynthesis identified from common DEGs.

Structural genes encode enzymes that directly participate in the biosynthesis of anthocyanin. From the pool of 503 common genes, we identified 7 structural genes involved in the anthocyanin biosynthesis pathway, namely *CHS* (T459_15321), *F3H* (T459_06228), *F3′5′H* (T459_28730), *DFR* (T459_06365), *ANS* (T459_00408), *UFGT* (T459_26178), and *3RT* (MSTRG.12525), as shown in Fig. 6. The expression levels of these genes were relatively higher in purple fruits (HN191 at 10, 20, 30DAA, HN192 at 30 DAA, HN005 at 30DAA) compared to green fruits (EJT at 10, 20, 30 DAA). Moreover, their expression in purple fruits sharply decreased at 60 DAA, aligning with the observed pattern of anthocyanin accumulation. An additional important structural gene, *CHI* (T459_15095), was not part of the pool of 503 common DEGs because its fold change between HN191 and EJT at 10 DAA did not meet the threshold of 2. Nevertheless, in the remaining four comparisons, the expression levels of *CHI* in purple fruits were more than two times higher than in green fruits. For further reference, the sequences of these 8 structural genes can be found in Table S6.

### TF prediction from common DEGs

**Figure 7:**
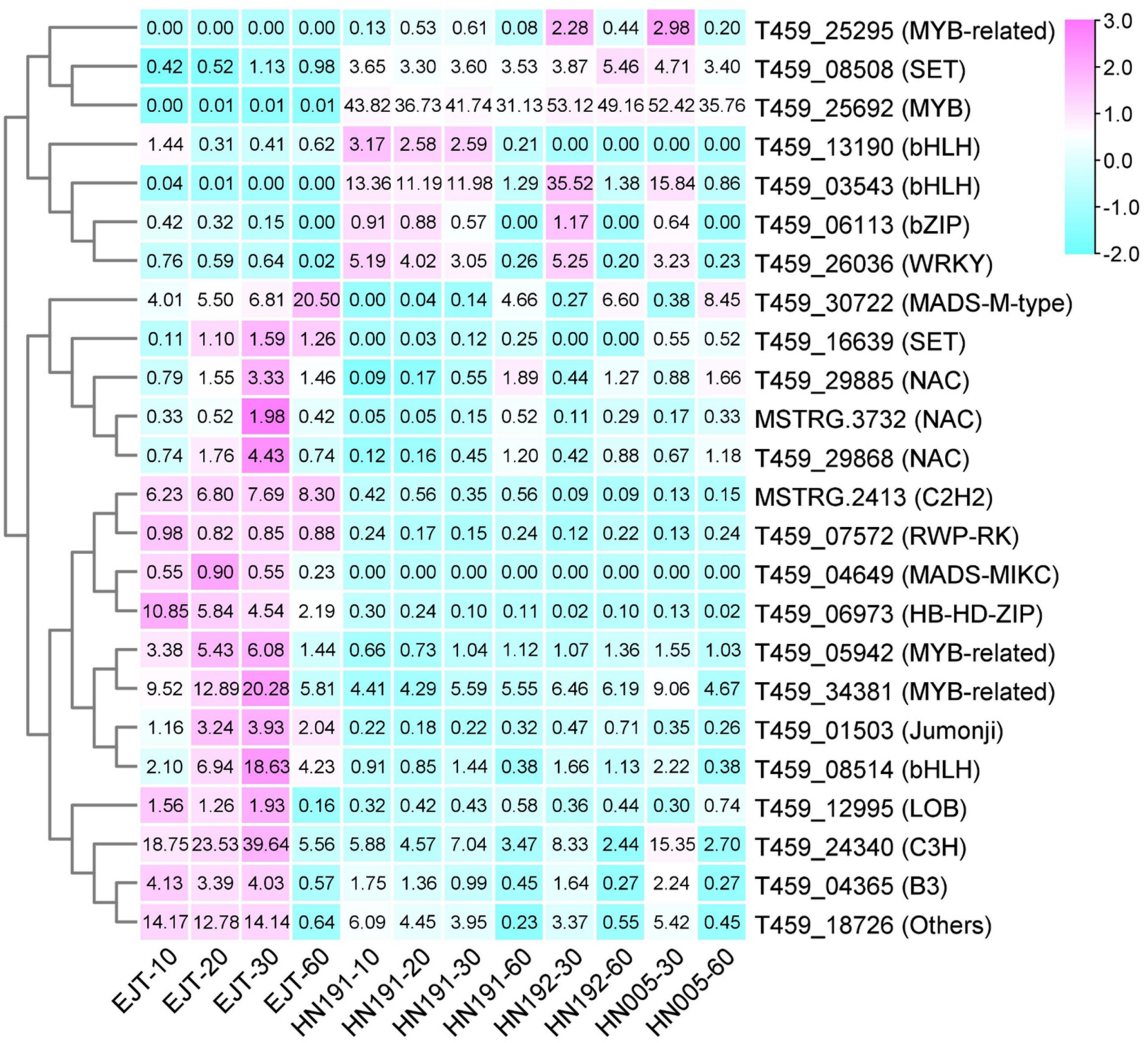
Heatmap of transcription factors (TFs) predicted from common DEGs.

Regulatory genes are responsible for encoding transcription factors that play a crucial role in the biosynthesis of anthocyanin (Lloyd et al., 2017). To identify these transcription factors, we utilized the iTAK software to predict them from the 503 common DEGs. As a result, a total of 24 transcription factors were identified (Table S7). We then generated a heatmap (Fig. 7) based on the FPKM values of these 24 transcription factors. Based on their expression patterns, these transcription factors can be divided into two distinct groups. The first group consisted of seven transcription factors (from T459_25295 to T459_26036) that exhibited relatively low expression in green fruits (EJT at 10, 20, 30 DAA), but showed higher expression in fruits of the three purple varieties (HN191 at 10, 20, 30 DAA, HN192 at 30 DAA, HN005 at 30 DAA). Conversely, the second group included 17 transcription factors (from T459_30722 to T459_18726) that displayed the opposite expression pattern. These findings suggest that the seven transcription factors in the first group may be closely associated with the biosynthesis of anthocyanin in pepper fruit.

### qPCR validation

To validate the reliability of the RNA-seq data, we selected and analyzed 10 structural genes and 11 regulatory genes (transcription factors) associated with anthocyanin biosynthesis using qPCR.

**Figure 8:**
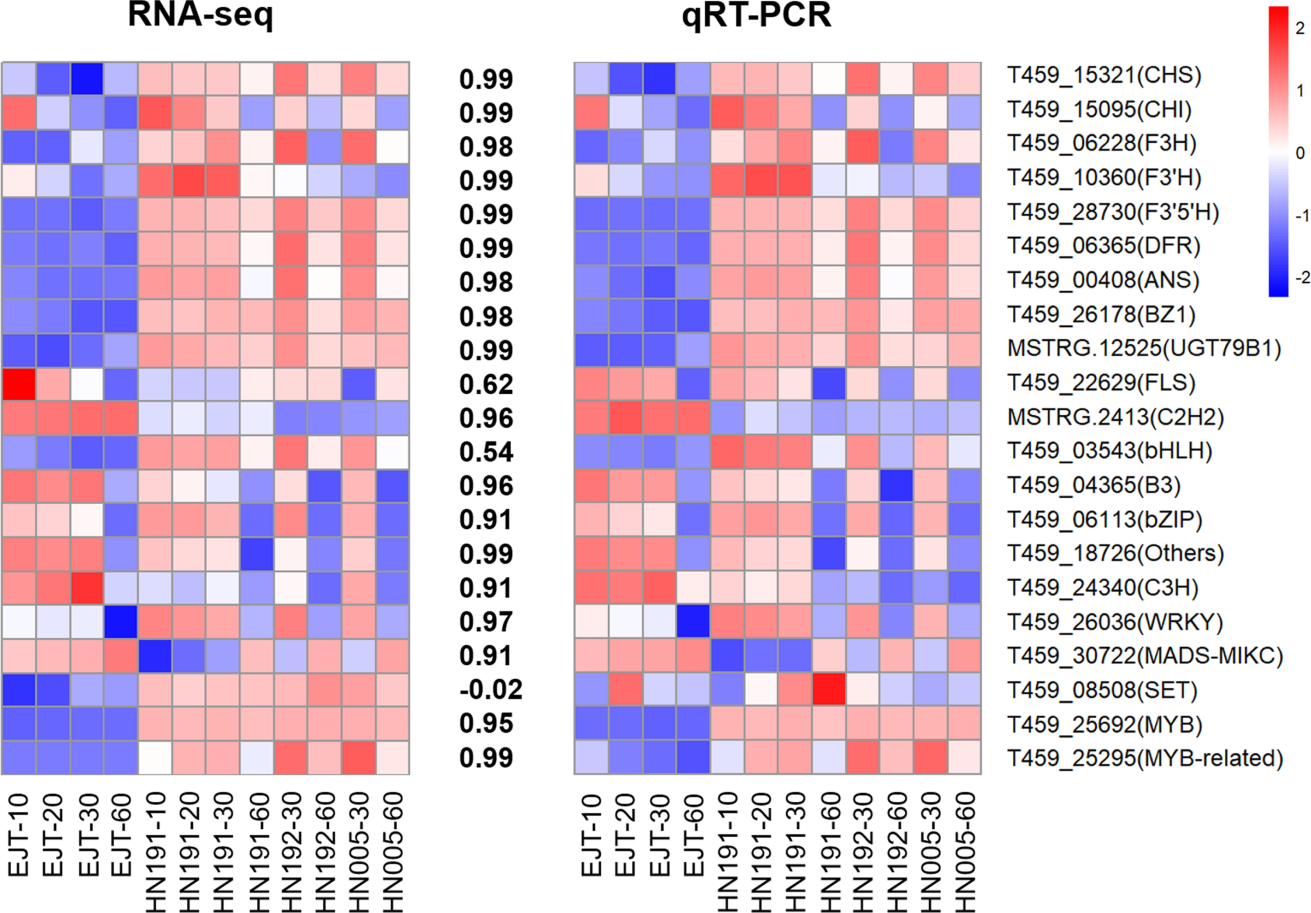
Validation of expression levels of selected genes in four pepper varieties. The heatmaps represent expression profiles of selected genes (labeled on the right side). The left heatmap is derived from RNA-seq analysis, and the right heatmap is derived from qRT-PCR analysis. The color scale represents the Z-score. The values between the two heatmaps represent the correlation coefficient between the expression data of selected genes obtained from RNA-seq and qRT-PCR analyses.

The qPCR results demonstrated that the expression patterns of these selected genes were largely consistent with those observed in the RNA-seq data, except for one gene T459_08508 (Fig. 8, Table S8, and Table S9). The Pearson correlation coefficient between the RNA-seq and qRT-PCR data ranged from 0.54 to 0.99, with an average value of 0.88. This suggests that the RNA-seq data accurately reflect the abundance of transcript levels.

## Discussion

Transcriptome refers to the complete set of transcripts in a cell, specific to a particular physiological condition or developmental stage (Wang, Gerstein & Snyder, 2009). Various technologies have been developed to study the transcriptome, including hybridization-based microarrays, Sanger sequencing-based methods, and RNA sequencing (Yamada et al., 2003; Cheung et al., 2008; Wang, Gerstein & Snyder, 2009). RNA sequencing, also known as RNA-Seq, utilizes the Next Generation Sequencing (NGS) platform and has become a powerful tool for identifying differentially expressed genes (DEGs) and potential molecular mechanisms (Vogel et al., 2016; Wei et al., 2017; Yang et al., 2019). In recent years, RNA-Seq has been extensively applied in comparative transcriptome analyses of green and purple fruit peppers (Liu et al., 2020b; Tang et al., 2020; Meng et al., 2022).

Unfortunately, RNA-seq often yields a large number of differentially expressed genes (DEGs), making it challenging to identify the specific genes that control a particular phenotype. In this study, we aimed to address this issue by comparing the transcriptomes of three purple-fruited pepper varieties (HN191, HN192, and HN005) with that of one green-fruited variety (EJT) at 30 days after anthesis (DAA). Additionally, we compared the transcriptomes of HN191 and EJT at two earlier developmental stages (10 and 20 DAA). Hence, in total, we conducted five comparisons between green and purple fruit. From these comparisons, we identified a set of 503 common DEGs.

To further narrow down the list of candidate genes, we also utilized transcriptome data from the ripe stage (60 DAA) of four varieties. This selection was based on the understanding that anthocyanins tend to degrade in ripe fruits, leading to a decrease in the expression levels of genes involved in anthocyanin biosynthesis (Aza-González et al., 2013; Borovsky et al., 2004). By focusing on this stage, we aimed to significantly reduce the number of candidate genes.

Among the 503 common DEGs, we identified 7 structural genes involved in anthocyanin biosynthesis. These genes included *CHS* (T459_15321), *F3H* (T459_06228), *F3′5′H* (T459_28730), *DFR* (T459_06365), *ANS* (T459_00408), *UFGT* (T459_26178), and a novel candidate gene designated as *3RT* (MSTRG.12525). Another structural gene, *CHI* (T459_15095), was not grouped in the 503 common DEGs because its fold change between HN191 and EJT at 10 DAA did not surpass the threshold of 2. The expression levels of these structural genes were relatively low in green fruits (specifically, EJT at 10, 20, and 30 DAA), but exhibited higher expression in purple fruits (specifically, HN191 at 10, 20, and 30 DAA; HN192 and HN005 at 30 DAA), which is consistent with previous studies (Tang et al., 2020; Jung et al., 2019). The differential gene expression of the structural genes, especially LBGs, is the reason for anthocyanin production (Tang et al., 2020). Therefore, these findings suggest that the upregulation of these structural genes is responsible for anthocyanin biosynthesis in purple pepper fruits.

Notably, MSTRG.12525 was identified as a novel structural gene involved in anthocyanin biosynthesis in this study. Blast analysis indicated that the MSTRG.12525 gene shares 99.68% sequence identity with *Capsicum annuum* anthocyanidin-3-O-glucoside rhamnosyltransferase (LOC107861697), which is known to control the conversion of anthocyanidin-3-O-glucosides to anthocyanidin-3-O-rutinosides. Previous research has shown that the predominant anthocyanins accumulated in purple pepper fruits are delphinidin 3-O-rutinoside, delphinidin 3-p-coumaroyl-rutinoside-5-glucoside, and delphinidin 3-cis-coumaroylrutinoside-5-glucoside (Meng et al., 2022; Tang et al., 2020). Thus, it could be inferred that MSTRG.12525 might play a role in the conversion of 3-O-glucoside to 3-O-rutinoside in purple pepper fruits.

Among the 503 common DEGs, a total of 24 transcription factors were identified, seven of which exhibited significantly higher expression in purple fruits (specifically, HN191 at 10, 20, and 30 DAA; HN192 and HN005 at 30 DAA) than in green fruits. However, their expression levels decreased in ripe fruits (60 DAA), which correlates with the pattern of anthocyanin accumulation. These findings, suggest that these TFs may play a role in the biosynthesis of anthocyanin.

For gene T459_08508, the expression correlation coefficient between RNA-seq and qRT-PCR was −0.02. As for the gene T459_13190, it encodes a bHLH transcription factor. Its expression level was higher in the unripe fruit of HN191 compared to EJT. However, its expression levels were relatively low in the unripe fruit of HN192 and HN005 at 30 DAA. Therefore, based on these observations, it is likely that these two genes may not be considered as candidate genes.

For the remaining five genes, two of them have already been reported. T459_25692 encodes an MYB transcription factor that is homologous to the *A* gene (GenBank accession number AJ608992.1). It is also known as *CaAN2* since it encodes a homolog of *Petunia anthocyanin 2* (*An2*) (Borovsky et al., 2004). Its expression has been detected throughout all stages of fruit development in the purple-fruit variety 5226, but not in the green-fruit variety PI 159234 (Borovsky et al., 2004). Overexpression of *CaAn2* in *Nicotiana benthamiana* and *Arabidopsis thaliana* resulted in the development of purple tissues (Jung et al., 2019). The promoter of *CaAn2* in purple C. annuum ’KC00134′plants contains an insertion of a non-long terminal repeat (LTR) retrotransposon called CanLTR-A, which may activate the expression of *CaAn2* by recruiting transcription factors at the 3′UTR (Jung et al., 2019). On the other hand, T459_03543 encodes a bHLH transcription factor that is homologous to AN1 (LOC107842687). This transcription factor, referred to as CaAN1, along with two other MYBs (CaANT1, LOC107854818; CaANT2, LOC107844901), were significantly upregulated in the purple pepper fruits (Tang et al., 2020). They can form an MBW complex with a WD40 protein (CaTTG1, LOC107862994), which is involved in the regulation of structural genes in the anthocyanin biosynthetic pathway (Tang et al., 2020).

However, the function of another three TFs in anthocyanin biosynthesis of pepper has not yet been reported. T459_25295 encodes a DIVARICATA-like (DIV) transcription factor, which belongs to the MYB-related family subgroup. Previous studies in chilli pepper (*CaDIV1*, *CaDIV3*, *CaDIV11*) have shown a positive correlation between DIVs and key genes of the flavonoid biosynthetic pathway (Arce-Rodríguez, Martínez & Ochoa-Alejo, 2021; Song et al., 2023). T459_06113 encodes a bZIP transcription factor. In pepper, a bZIP transcription factor CaHY5 has been found to play a crucial role in regulating anthocyanin accumulation in the hypocotyl. It achieves this by binding to the promoters of key genes involved in anthocyanin biosynthesis, including CaF3H, CaF3’5’H, CaDFR, CaANS, and CaGST (Chen et al., 2022). Additionally, research has shown that CaMYB113 can interact with CabHLH143 and CaHY5, suggesting a collaborative role of these three genes in UV-B-induced anthocyanin biosynthesis in pepper fruit (Wang et al., 2022). In tomato, a similar bZIP transcription factor called SlHY5 has been found to directly recognize and bind to the promoters of anthocyanin biosynthesis genes, regulating the accumulation of anthocyanin (Liu et al., 2018a). Another bZIP transcription factor, MdbZIP44, in apple has been found to promote anthocyanin accumulation in response to ABA by enhancing the binding of MdMYB1 to the promoters of downstream target genes (An et al., 2018). T459_26036 encodes a WRKY transcription factor. Previous studies have reported that WRKY transcription factors can interact with MYB and promote anthocyanin biosynthesis in apple, pear, potato, and eggplant (An et al., 2019; Cong et al., 2021; Zhang et al., 2021; He et al., 2021). In summary, these three TFs may represent novel candidate genes that contribute to anthocyanin biosynthesis in pepper fruit.

## Conclusions

In this study, we investigated the transcriptome dynamics of three purple-fruited varieties (HN191, HN192, HN005) and one green-fruited variety (EJT) during fruit development using RNA-seq. A total of five comparisons between green and purple fruit have been analyzed and 503 common differentially expressed genes (DEGs) have been identified. Among these DEGs, eight structural genes involved in the anthocyanin biosynthesis pathway and 24 transcription factors (TFs) were identified. We hypothesize that one structural gene and three TFs could potentially be novel candidate genes for anthocyanin biosynthesis based on their expression patterns. These findings contribute to a comprehensive understanding of the mechanisms underlying anthocyanin biosynthesis in pepper and provide potential target genes for the genetic improvement of anthocyanin-rich pepper germplasm.

## Supplemental Information

10.7717/peerj.16792/supp-1 Supplemental Information 1 List of primers used in qPCR analysis The first column (from A2 to A23) indicates Gene ID of 22 selected genes (including the reference gene) for qPCR validation. The first row (from B1 to G1) indicates Forward primer, Reverse Primer, Product Size, Tm, Primer Blast result in NCBI, and Primer Blast result gene in NCBI, respectively.

10.7717/peerj.16792/supp-2 Supplemental Information 2 MIQE checklist for qPCR methods and analysis The first column indicates Item to check according to the MIQE guidelines. The second column indicates importance. E is short for essential while D is short for Desirable. The third column indicates Checklist in this study.

10.7717/peerj.16792/supp-3 Supplemental Information 3 RNA Quality assessment The first row (from B1 to G1) indicates Concentration, Volume, Yield, RIN (RNA Integrity Number), 28S/18S, and Quality Class of RNA, respectively. The first column (from A2 to A37) indicates 12 sequencing fruits (each contains 3 independent biological repeats, resulting in a total of 36 samples) in this study.

10.7717/peerj.16792/supp-4 Supplemental Information 4 Statistics of sequencing data The first row (from B1 to H1) indicates Raw Reads, Raw Base, Clean Reads, Clean Base, Q20 (quality score of 20, an error rate of 1 in 100), Q30 (quality score of 30, an error rate of 1 in 1000), GC content, respectively. The first column (from A2 to A37) indicates 12 sequencing fruits (each contains 3 independent biological repeats, resulting in a total of 36 samples) in this study. For numbers from B2 to E37, M means 10^6^ and G means 10^9^.

10.7717/peerj.16792/supp-5 Supplemental Information 5 Power analysis between different groups The first column (from A2 to A10) indicates different comparison groups. The first row (from B1 to G1) indicates parameters used for calculating Power values. The first row (from H1 to J1) indicates calculated Power values when effect =2, 3, and 4, respectively.

10.7717/peerj.16792/supp-6 Supplemental Information 6 List of 8 structure genes The first row (from B1 to M1) indicates 12 sequencing fruits (each represents the mean value of three independent biological repeats). The first row (from N1 to P1) indicates KEGG annotation, KEGG pathway, and sequence of novel candidate genes, respectively. The first column (from A2 to A9) indicates Gene ID of 8 structure genes. Numbers from B2 to M9 indicate FPKM values obtained from RNA-seq.

10.7717/peerj.16792/supp-7 Supplemental Information 7 List of 24 TFs The first row (from B1 to M1) indicates 12 sequencing fruits (each represents the mean value of three independent biological repeats). The first row (from N1 to Q1) indicates KEGG annotation, KEGG pathway, TF family, and sequence of novel candidate genes, respectively. The first column (from A2 to A25) indicates Gene ID of 24 TFs. Numbers from B2 to M25 indicate FPKM values obtained from RNA-seq.

10.7717/peerj.16792/supp-8 Supplemental Information 8 FPKM of 21 validated genes in all samples The first row (from B1 to V1) indicates gene ID. The first column (from A2 to A37) indicates 12 sequencing fruits (each contains 3 independent biological repeats, resulting in a total of 36 samples) in this study. Numbers from B2 to V37 indicate FPKM values obtained from RNA-seq.

10.7717/peerj.16792/supp-9 Supplemental Information 9 Raw qPCR data of 21 validated genes in all samples The first row (from B1 to V1) indicates gene ID. The first column (from A2 to A37) indicates 12 sequencing fruits (each contains 3 independent biological repeats, resulting in a total of 36 samples) in this study. Numbers from B2 to V37 indicate related expression levels calculated from Cq value of qPCR.

## Additional Information and Declarations

### Data Availability

The following information was supplied regarding data availability: The data is available at the CNGB Sequence Archive (CNSA) of the China National GeneBank DataBase (CNGBdb): CNP0004460.

## References

[ref-1] An JP, Yao JF, Xu RR, You CX, Wang XF, Hao YJ (2018). Apple bZIP transcription factor MdbZIP44 regulates abscisic acid-promoted anthocyanin accumulation. Plant, Cell & Environment.

[ref-2] An JP, Zhang XW, You CX, Bi SQ, Wang XF, Hao YJ (2019). MdWRKY40 promotes wounding-induced anthocyanin biosynthesis in association with MdMYB1 and undergoes MdBT2-mediated degradation. New Phytologist.

[ref-3] Arce-Rodríguez ML, Martínez O, Ochoa-Alejo N (2021). Genome-wide identification and analysis of the MYB transcription factor gene family in chili pepper (*Capsicum* spp.). International Journal of Molecular Sciences.

[ref-4] Aza-González C, Herrera-Isidrón L, Núñez Palenius HG, Martínez De La Vega O, Ochoa-Alejo N (2013). Anthocyanin accumulation and expression analysis of biosynthesis-related genes during chili pepper fruit development. Biologia Plantarum.

[ref-5] Borovsky Y, Michal OS, Rinat O, Walter DJ, Iaran I (2004). The A locus that controls anthocyanin accumulation in pepper encodes a MYB transcription factor homologous to Anthocyanin2 of Petunia. Theoretical and Applied Genetics.

[ref-6] Bustin SA, Benes V, Garson JA, Hellemans J, Huggett J, Kubista M, Mueller R, Nolan T, Pfaffl MW, Shipley GL, Vandesompele J, Wittwer CT (2009). The MIQE guidelines: minimum information for publication of quantitative real-time PCR experiments. Clinical Chemistry.

[ref-7] Chalker-Scott L (1999). Environmental significance of anthocyanins in plant stress responses. Photochemistry and Photobiology.

[ref-8] Chen C, Chen H, Zhang Y, Thomas HR, Xia R (2020). TBtools: an integrative toolkit developed for interactive analyses of big biological data. Molecular Plant.

[ref-9] Chen R, Yang C, Gao H, Shi C, Zhang Z, Lu G, Shen X, Tang Y, Li F, Lu Y, Ouyang B (2022). Induced mutation in ELONGATED HYPOCOTYL5 abolishes anthocyanin accumulation in the hypocotyl of pepper. Theoretical and Applied Genetics.

[ref-10] Chen S, Zhou Y, Chen Y, Gu J (2018). fastp: an ultra-fast all-in-one FASTQ preprocessor. Bioinformatics.

[ref-11] Cheung F, Win J, Lang JM, Hamilton J, Vuong H, Leach JE, Kamoun S, Lévesque CA, Tisserat N, Buell CR (2008). Analysis of the Pythium ultimum transcriptome using Sanger and Pyrosequencing approaches. BMC Genomics.

[ref-12] Cong L, Qu Y, Sha G, Zhang S, Ma Y, Chen M, Zhai R, Yang C, Xu L, Wang Z (2021). PbWRKY75 promotes anthocyanin synthesis by activating PbDFR, PbUFGT, and PbMYB10b in pear. Physiologia Plantarum.

[ref-13] De Pascual-Teresa S, Moreno DA, García-Viguera C (2010). Flavanols and anthocyanins in cardiovascular health: a review of current evidence. International Journal of Molecular Sciences.

[ref-14] Gonzali S, Mazzucato A, Perata P (2009). Purple as a tomato: towards high anthocyanin tomatoes. Trends in Plant Science.

[ref-15] Gould KS (2004). Nature’s swiss army knife: the diverse protective roles of anthocyanins in leaves. Journal of Biomedicine & Biotechnology.

[ref-16] Hart SN, Therneau TM, Zhang Y, Poland GA, Kocher JP (2013). Calculating sample size estimates for RNA sequencing data. Journal of Computational Biology.

[ref-17] He Y, Wang Z, Ge H, Liu Y, Chen H (2021). Weighted gene co-expression network analysis identifies genes related to anthocyanin biosynthesis and functional verification of hub gene SmWRKY44. Plant Science.

[ref-18] Holton TA, Cornish EC (1995). Genetics and biochemistry of anthocyanin biosynthesis. The Plant Cell.

[ref-19] Jung S, Venkatesh J, Kang MY, Kwon JK, Kang BC (2019). A non-LTR retrotransposon activates anthocyanin biosynthesis by regulating a MYB transcription factor in *Capsicum annuum*. Plant Science.

[ref-20] Kim D, Langmead B, Salzberg SL (2015). HISAT: a fast spliced aligner with low memory requirements. Nature Methods.

[ref-21] Kim S, Park M, Yeom SI, Kim YM, Lee JM, Lee HA, Seo E, Choi J, Cheong K, Kim KT, Jung K, Lee GW, Oh SK, Bae C, Kim SB, Lee HY, Kim SY, Kim MS, Kang BC, Jo YD, Yang HB, Jeong HJ, Kang WH, Kwon JK, Shin C, Lim JY, Park JH, Huh JH, Kim JS, Kim BD, Cohen O, Paran I, Suh MC, Lee SB, Kim YK, Shin Y, Noh SJ, Park J, Seo YS, Kwon SY, Kim HA, Park JM, Kim HJ, Choi SB, Bosland PW, Reeves G, Jo SH, Lee BW, Cho HT, Choi HS, Lee MS, Yu Y, Choi YDo, Park BS, van Deynze A, Ashrafi H, Hill T, Kim WT, Pai HS, Ahn HK, Yeam I, Giovannoni JJ, Rose JK, Sørensen I, Lee SJ, Kim RW, Choi IY, Choi BS, Lim JS, Lee YH, Choi D (2014). Genome sequence of the hot pepper provides insights into the evolution of pungency in *Capsicum* species. Nature Genetics.

[ref-22] Lamy S, Lafleur R, Bédard V, Moghrabi A, Barrette S, Gingras D, Béliveau R (2007). Anthocyanidins inhibit migration of glioblastoma cells: structure—activity relationship and involvement of the plasminolytic system. Journal of Cellular Biochemistry.

[ref-23] Lightbourn GJ, Griesbach RJ, Novotny JA, Clevidence BA, Rao DD, Stommel JR (2008). Effects of anthocyanin and carotenoid combinations on foliage and immature fruit color of *Capsicum annuum* L. Journal of Heredity.

[ref-24] Liu J, Ai X, Wang Y, Lu Q, Li T, Wu L, Sun L, Shen H (2020a). Fine mapping of the *Ca3GT* gene controlling anthocyanin biosynthesis in mature unripe fruit of *Capsicum annuum* L. Theoretical and Applied Genetics.

[ref-25] Liu CC, Chi C, Jin LJ, Zhu J, Yu JQ, Zhou YH (2018a). The bZip transcription factor HY5 mediates CRY1a-induced anthocyanin biosynthesis in tomato. Plant, Cell & Environment.

[ref-26] Liu Y, Lv J, Liu Z, Wang J, Yang B, Chen W, Ou L, Dai X, Zhang Z, Zou X (2020b). Integrative analysis of metabolome and transcriptome reveals the mechanism of color formation in pepper fruit (*Capsicum annuum* L.). Food Chemistry.

[ref-27] Liu Y, Tikunov Y, Schouten RE, Marcelis L, Visser R, Bovy A (2018b). Anthocyanin biosynthesis and degradation mechanisms in solanaceous vegetables: a review. Frontiers in Chemistry.

[ref-28] Lloyd A, Brockman A, Aguirre L, Campbell A, Bean A, Cantero A, Gonzalez A (2017). Advances in the MYB-bHLH-WD repeat (MBW) pigment regulatory model: addition of a WRKY factor and co-option of an anthocyanin MYB for betalain regulation. Plant and Cell Physiology.

[ref-29] Meng Y, Zhang H, Fan Y, Yan L (2022). Anthocyanins accumulation analysis of correlated genes by metabolome and transcriptome in green and purple peppers (*Capsicum annuum*). BMC Plant Biology.

[ref-30] Pertea M, Kim D, Pertea GM, Leek JT, Salzberg SL (2016). Transcript-level expression analysis of RNA-seq experiments with HISAT, StringTie and Ballgown. Nature Protocols.

[ref-31] Peterson PA (1959). Linkage of fruit shape and color genes in *Capsicum*. Genetics.

[ref-32] Petroni K, Tonelli C (2011). Recent advances on the regulation of anthocyanin synthesis in reproductive organs. Plant Science.

[ref-33] Reddy MK, Alexander-Lindo RL, Nair MG (2005). Relative inhibition of lipid peroxidation, cyclooxygenase enzymes, and human tumor cell proliferation by natural food colors. Journal of Agricultural and Food Chemistry.

[ref-34] Song J, Sun B, Chen C, Ning Z, Zhang S, Cai Y, Zheng X, Cao B, Chen G, Jin D, Li B, Bian J, Lei J, He H, Zhu Z (2023). An R-R-type MYB transcription factor promotes non-climacteric pepper fruit carotenoid pigment biosynthesis. Plant Journal.

[ref-35] Steyn WJ, Wand SJE, Holcroft DM, Jacobs G (2002). Anthocyanins in vegetative tissues: a proposed unified function in photoprotection. New Phytologist.

[ref-36] Stommel JR, Lightbourn GJ, Winkel BS, Griesbach RJ (2009). Transcription factor families regulate the anthocyanin biosynthetic pathway in *Capsicum annuum*. Journal of the American Society for Horticultural Science.

[ref-37] Tanaka Y, Sasaki N, Ohmiya A (2008). Biosynthesis of plant pigments: anthocyanins, betalains, and carotenoids. The Plant Journal.

[ref-38] Tang B, Li L, Hu Z, Chen Y, Tan T, Jia Y, Xie Q, Chen G (2020). Anthocyanin accumulation and transcriptional regulation of anthocyanin biosynthesis in purple pepper. Journal of Agricultural and Food Chemistry.

[ref-39] Varet H, Brillet-Guéguen L, Coppée JY, Dillies MA (2016). SARTools: a DESeq2- and EdgeR-based R pipeline for comprehensive differential analysis of RNA-Seq data. PLOS ONE.

[ref-40] Vogel C, Bodenhausen N, Gruissem W, Vorholt JA (2016). The *Arabidopsis* leaf transcriptome reveals distinct but also overlapping responses to colonization by phyllosphere commensals and pathogen infection with impact on plant health. New Phytologist.

[ref-41] Wang G, Chen B, Du H, Zhang F, Zhang H, Wang Y, He H, Geng S, Zhang X (2018). Genetic mapping of anthocyanin accumulation-related genes in pepper fruits using a combination of SLAF-seq and BSA. PLOS ONE.

[ref-42] Wang Z, Gerstein M, Snyder M (2009). RNA-Seq: a revolutionary tool for transcriptomics. Nature Reviews Genetics.

[ref-43] Wang Y, Liu S, Wang H, Zhang Y, Li W, Liu J, Cheng Q, Sun L, Shen H (2022). Identification of the regulatory genes of UV-B-induced anthocyanin biosynthesis in pepper fruit. International Journal of Molecular Sciences.

[ref-44] Wei Q, Jiao C, Guo L, Ding YL, Cao JJ, Feng JY, Dong XB, Mao LY, Sun HH, Yu F, Yang GY, Shi PJ, Ren GD, Fei ZJ (2017). Exploring key cellular processes and candidate genes regulating the primary thickening growth of *Moso* underground shoots. New Phytologist.

[ref-45] Yamada K, Lim J, Dale J, Chen H, Shinn P, Palm CJ, Southwick AM (2003). Empirical analysis of transcriptional activity in the *Arabidopsis* genome. Science.

[ref-46] Yang F, Zhu G, Wei Y, Gao J, Liang G, Peng L, Lu C, Jin J (2019). Low-temperature-induced changes in the transcriptome reveal a major role of CgSVP genes in regulating flowering of Cymbidium goeringii. BMC Genomics.

[ref-47] Zhang Y, Butelli E, Martin C (2014). Engineering anthocyanin biosynthesis in plants. Current Opinion in Plant Biology.

[ref-48] Zhang Z, Li DW, Jin JH, Yin YX, Zhang HX, Chai WG, Gong ZH (2015). VIGS approach reveals the modulation of anthocyanin biosynthetic genes by CaMYB in chili pepper leaves. Frontiers in Plant Science.

[ref-49] Zhang H, Zhang Z, Zhao Y, Guo D, Zhao X, Gao W, Cahill D (2021). StWRKY13 promotes anthocyanin biosynthesis in potato (Solanum tuberosum) tubers. Functional Plant Biology.

[ref-50] Zheng Y, Jiao C, Sun H, Rosli H, Pombo MA, Zhang P, Banf M, Dai X, Martin GB, Giovannoni JJ, Zhao PX, Rhee SY, Fei Z (2016). iTAK: a program for genome-wide prediction and classification of plant transcription factors, transcriptional regulators, and protein kinases. Molecular Plant.

